# 3-(4-Fluoro­phen­yl)-2-(4-methoxy­phen­oxy)-4-oxo-5-phenyl-4,5-dihydro-3*H*-pyrrolo[3,2-*d*]pyrimidine-7-carbonitrile

**DOI:** 10.1107/S1600536808024410

**Published:** 2008-08-06

**Authors:** Guo-Ping Zeng, Shi-Rong Yan

**Affiliations:** a Department of the Clinical Laboratory, Shiyan People’s Hospital Affiliated to Yunyang Medical College, Shiyan 442000, People’s Republic of China; b Department of Chemistry and Life Science, Hubei University of Education, Wuhan, 430205, People’s Republic of China

## Abstract

There are two crystallographically independent mol­ecules in the asymmetric unit of the title compound, C_26_H_17_FN_4_O_3_, which differ in the dihedral angles between the aromatic rings (fluorophenyl, phenyl) and the pyrrolopyrimidine rings [0.6 (3)/76.3° and 73.7 (3)/64.6°, respectively]. The crystal structure is mainly stabilized by C—H⋯O and C—H⋯F inter­actions.

## Related literature

For related preparation and biological activity, see: Shih *et al.* (2002 ▶); Niwas *et al.* (1994 ▶). For related literature, see: Ding *et al.* (2004 ▶).
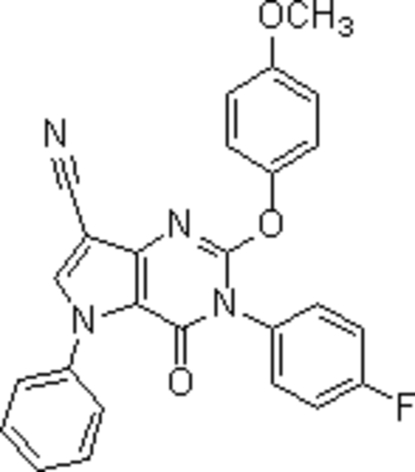


## Experimental

### 

#### Crystal data

C_26_H_17_FN_4_O_3_
*M* *_r_* = 452.44
Tetragonal, 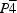

*a* = 17.7893 (14) Å
*c* = 14.332 (12) Å
*V* = 4536 (1) Å^3^
*Z* = 8
Mo *K*α radiation
μ = 0.10 mm^−1^
*T* = 298 (2) K
0.36 × 0.23 × 0.20 mm

#### Data collection

Bruker SMART 4K CCD area-detector diffractometer
Absorption correction: multi-scan (*SADABS*; Sheldrick, 2003 ▶) *T*_min_ = 0.967, *T*_max_ = 0.981
29534 measured reflections
4649 independent reflections
2383 reflections with *I* > 2σ(*I*)
*R*_int_ = 0.136

#### Refinement

*R*[*F* ^2^ > 2σ(*F* ^2^)] = 0.054
*wR*(*F* ^2^) = 0.087
*S* = 0.89
4649 reflections
615 parameters
H-atom parameters constrained
Δρ_max_ = 0.18 e Å^−3^
Δρ_min_ = −0.13 e Å^−3^

### 

Data collection: *SMART* (Bruker, 2001 ▶); cell refinement: *SAINT-Plus* (Bruker, 2001 ▶); data reduction: *SAINT-Plus*; program(s) used to solve structure: *SHELXS97* (Sheldrick, 2008 ▶); program(s) used to refine structure: *SHELXL97* (Sheldrick, 2008 ▶); molecular graphics: *PLATON* (Spek, 2003 ▶); software used to prepare material for publication: *SHELXTL* (Sheldrick, 2008 ▶).

## Supplementary Material

Crystal structure: contains datablocks I, global. DOI: 10.1107/S1600536808024410/bt2753sup1.cif

Structure factors: contains datablocks I. DOI: 10.1107/S1600536808024410/bt2753Isup2.hkl

Additional supplementary materials:  crystallographic information; 3D view; checkCIF report

## Figures and Tables

**Table 1:** Hydrogen-bond geometry (Å, °)

*D*—H⋯*A*	*D*—H	H⋯*A*	*D*⋯*A*	*D*—H⋯*A*
C19—H19⋯O6	0.93	2.57	3.421 (8)	152
C38—H38⋯O2^i^	0.93	2.61	3.405 (8)	144
C50—H50*B*⋯F1^ii^	0.96	2.51	3.407 (7)	155
C15—H15⋯O1^iii^	0.93	2.41	3.201 (9)	143

Symmetry codes: (i) 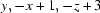; (ii) 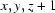; (iii) 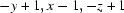.

**Fig. 1.**
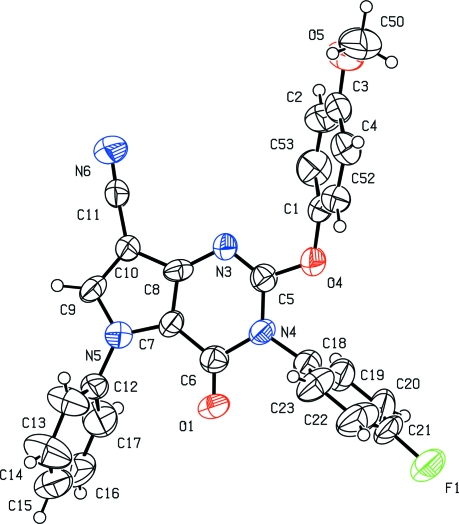
The molecular structure of the two molecules of title compound, showing the atom-labeling scheme. Displacement ellipsoids are drawn at the 50% probability level.

## supplementary crystallographic information

### Comment

Heterocyclic compounds containing a fused pyrimidinone system have various applications in agriculture and exhibit remarkable biological activity (Ding *et al.*, 2004). Pyrrolopyrimidine derivatives are of great importance because of their remarkable biological properties (Shih, *et al.*, 2002; Niwas, *et al.*, 1994). We present here the crystal structure of the title compound, (Fig. 1), which can be used as a precursor for obtaining bioactive molecules. Within the molecule of the title compound, the bond lengths and angles present no unusual features. In the crystal stucture, two crystallographically independent molecules are found in the asymmetric unit. The mean planes of the pyrrolo[3,2-*d*]pyrimidine ring system [maximum deviation of 0.028 (4)Å and -0.044 Å for atom C31 and C28, respectively] (A), and the fluorophenyl (B) and phenyl (C12–17 and C35–40) rings (C) form dihedral angles of A/B=80.6 (3)/76.3° and A/C=73.7 (3)/64.6°, respectively. The crystal packing is mainly stabilized by C—H···O and C—H···F   interactions (Table 1, Fig.2).

### Experimental

The title compound was obtained in excellent yield via aza-Wittig reaction. Crystals suitable for single-crystal X-ray diffraction were obtained by recrystallization from a mixed solvent of ethanol and dichloromethane (1:2 v/v) at room temperature.

### Refinement

All H-atoms were positioned geometrically and refined using a riding model with C—H = 0.93 Å, *U*_iso_ = 1.2*U*_eq_ (C) for C*sp*^2^, C—H = 0.96 Å, *U*_iso_ = 1.5*U*_eq_ (C) for CH_3_. The methyl groups were allowed to rotate but not to tip.

### Crystal data

**Table:**

C_26_H_17_FN_4_O_3_	*D*_x_ = 1.325 Mg m^−^^3^
*M**_r_* = 452.44	Mo *K*α radiation, λ = 0.71073 Å
Tetragonal, *P*4	Cell parameters from 1922 reflections
Hall symbol: P -4	θ = 2.7–15.1°
*a* = 17.7893 (14) Å	µ = 0.10 mm^−^^1^
*c* = 14.332 (12) Å	*T* = 298 K
*V* = 4536 (1) Å^3^	Block, colorless
*Z* = 8	0.36 × 0.23 × 0.20 mm
*F*(000) = 1872	

### Data collection

**Table:**

Bruker SMART 4K CCD area-detector diffractometer	4649 independent reflections
Radiation source: fine-focus sealed tube	2383 reflections with *I* > 2σ(*I*)
graphite	*R*_int_ = 0.136
φ and ω scans	θ_max_ = 26.0°, θ_min_ = 1.8°
Absorption correction: multi-scan (*SADABS*; Sheldrick, 2003)	*h* = −21→21
*T*_min_ = 0.967, *T*_max_ = 0.981	*k* = −14→21
29534 measured reflections	*l* = −15→17

### Refinement

**Table:**

Refinement on *F*^2^	Primary atom site location: structure-invariant direct methods
Least-squares matrix: full	Secondary atom site location: difference Fourier map
*R*[*F*^2^ > 2σ(*F*^2^)] = 0.055	Hydrogen site location: inferred from neighbouring sites
*wR*(*F*^2^) = 0.087	H-atom parameters constrained
*S* = 0.89	*w* = 1/[σ^2^(*F*_o_^2^) + (0.0193*P*)^2^] where *P* = (*F*_o_^2^ + 2*F*_c_^2^)/3
4649 reflections	(Δ/σ)_max_ = 0.001
615 parameters	Δρ_max_ = 0.18 e Å^−^^3^
0 restraints	Δρ_min_ = −0.13 e Å^−^^3^

### Special details

**Table:**

Geometry. All e.s.d.'s (except the e.s.d. in the dihedral angle between two l.s. planes) are estimated using the full covariance matrix. The cell e.s.d.'s are taken into account individually in the estimation of e.s.d.'s in distances, angles and torsion angles; correlations between e.s.d.'s in cell parameters are only used when they are defined by crystal symmetry. An approximate (isotropic) treatment of cell e.s.d.'s is used for estimating e.s.d.'s involving l.s. planes.
Refinement. Refinement of *F*^2^ against ALL reflections. The weighted *R*-factor *wR* and goodness of fit *S* are based on *F*^2^, conventional *R*-factors *R* are based on *F*, with *F* set to zero for negative *F*^2^. The threshold expression of *F*^2^ > σ(*F*^2^) is used only for calculating *R*-factors(gt) *etc*. and is not relevant to the choice of reflections for refinement. *R*-factors based on *F*^2^ are statistically about twice as large as those based on *F*, and *R*- factors based on ALL data will be even larger.

### Fractional atomic coordinates and isotropic or equivalent isotropic displacement parameters (Å^2^)

**Table:**

	*x*	*y*	*z*	*U*_iso_*/*U*_eq_	
C31	0.6200 (3)	0.3475 (2)	1.0850 (3)	0.0412 (12)	
O2	0.66811 (18)	0.3679 (2)	1.3288 (3)	0.0630 (11)	
N7	0.7396 (2)	0.3638 (2)	1.1952 (3)	0.0412 (10)	
C28	0.7437 (3)	0.3632 (3)	1.0988 (4)	0.0444 (13)	
N9	0.5317 (2)	0.3477 (2)	1.1964 (3)	0.0477 (11)	
N8	0.6890 (2)	0.3529 (2)	1.0423 (3)	0.0420 (10)	
N4	1.0211 (2)	0.2597 (2)	0.7835 (3)	0.0494 (11)	
O3	0.81483 (18)	0.37435 (19)	1.0689 (2)	0.0553 (10)	
O4	1.06797 (19)	0.33069 (19)	0.8982 (2)	0.0640 (11)	
O1	0.9751 (2)	0.1919 (2)	0.6606 (3)	0.0789 (13)	
C34	0.5346 (3)	0.3317 (3)	0.9458 (4)	0.0581 (16)	
N10	0.5252 (2)	0.3231 (3)	0.8671 (4)	0.0746 (15)	
C26	0.8505 (3)	0.4065 (4)	0.7843 (4)	0.0609 (16)	
C5	1.0216 (3)	0.2733 (3)	0.8782 (4)	0.0512 (14)	
N5	0.8849 (2)	0.1032 (2)	0.8045 (3)	0.0567 (12)	
C6	0.9765 (3)	0.2016 (3)	0.7437 (4)	0.0544 (15)	
C11	0.8746 (3)	0.1359 (3)	1.0535 (5)	0.0606 (17)	
C24	0.8248 (3)	0.3848 (3)	0.9715 (4)	0.0491 (14)	
C7	0.9358 (3)	0.1621 (3)	0.8147 (4)	0.0501 (14)	
C1	1.0898 (3)	0.3401 (3)	0.9930 (4)	0.0514 (14)	
C25	0.8316 (3)	0.3370 (3)	0.8180 (4)	0.0604 (16)	
H25	0.8269	0.2967	0.7769	0.073*	
N3	0.9855 (2)	0.2373 (2)	0.9408 (3)	0.0471 (11)	
C33	0.5488 (3)	0.3395 (3)	1.0413 (3)	0.0454 (13)	
C30	0.6091 (3)	0.3514 (2)	1.1806 (3)	0.0413 (13)	
C29	0.6700 (3)	0.3619 (3)	1.2437 (4)	0.0460 (13)	
C55	0.8445 (3)	0.4541 (3)	0.9404 (4)	0.0648 (16)	
H55	0.8488	0.4939	0.9821	0.078*	
O5	1.1558 (3)	0.3706 (2)	1.2655 (3)	0.0924 (14)	
O6	0.8632 (2)	0.4231 (2)	0.6920 (3)	0.0884 (13)	
C40	0.4465 (3)	0.4174 (3)	1.2950 (4)	0.0758 (18)	
H40	0.4409	0.4520	1.2469	0.091*	
F2	0.98805 (19)	0.4048 (2)	1.4194 (3)	0.1178 (15)	
C18	1.0680 (3)	0.3002 (3)	0.7195 (4)	0.0556 (15)	
N6	0.8625 (3)	0.1418 (3)	1.1308 (4)	0.097 (2)	
C32	0.4970 (3)	0.3405 (3)	1.1138 (4)	0.0556 (15)	
H32	0.4452	0.3367	1.1061	0.067*	
C12	0.8670 (3)	0.0632 (3)	0.7187 (4)	0.0537 (14)	
C41	0.8059 (3)	0.3745 (3)	1.2517 (3)	0.0452 (13)	
C35	0.4932 (3)	0.3573 (3)	1.2841 (4)	0.0471 (14)	
C3	1.1378 (4)	0.3599 (3)	1.1729 (5)	0.0660 (17)	
C10	0.8910 (3)	0.1325 (3)	0.9557 (4)	0.0518 (14)	
C13	0.8993 (4)	−0.0050 (4)	0.7072 (5)	0.091 (2)	
H13	0.9283	−0.0260	0.7545	0.109*	
C16	0.8110 (4)	0.0538 (5)	0.5674 (5)	0.100 (2)	
H16	0.7803	0.0729	0.5205	0.120*	
C8	0.9412 (3)	0.1813 (3)	0.9075 (3)	0.0481 (14)	
C52	1.1609 (3)	0.3226 (3)	1.0168 (4)	0.0602 (16)	
H52	1.1933	0.3028	0.9723	0.072*	
C45	0.9151 (3)	0.3271 (4)	1.3238 (5)	0.088 (2)	
H45	0.9478	0.2874	1.3356	0.106*	
C19	1.0473 (4)	0.3678 (3)	0.6846 (4)	0.0735 (18)	
H19	1.0036	0.3903	0.7068	0.088*	
F1	1.1930 (2)	0.4033 (3)	0.5191 (2)	0.1469 (19)	
C36	0.5012 (3)	0.3064 (3)	1.3546 (4)	0.0623 (16)	
H36	0.5330	0.2653	1.3476	0.075*	
C9	0.8584 (3)	0.0860 (3)	0.8900 (4)	0.0588 (16)	
H9	0.8234	0.0486	0.9029	0.071*	
C38	0.4152 (3)	0.3771 (4)	1.4479 (5)	0.080 (2)	
H38	0.3891	0.3842	1.5034	0.096*	
C39	0.4081 (3)	0.4267 (4)	1.3765 (5)	0.090 (2)	
H39	0.3763	0.4678	1.3833	0.108*	
C46	0.8541 (3)	0.3167 (3)	1.2673 (4)	0.0652 (17)	
H46	0.8456	0.2702	1.2397	0.078*	
C43	0.8836 (4)	0.4536 (4)	1.3457 (4)	0.081 (2)	
H43	0.8942	0.5004	1.3715	0.098*	
C54	0.8196 (3)	0.3259 (3)	0.9119 (4)	0.0557 (15)	
H54	0.8079	0.2783	0.9344	0.067*	
C21	1.1513 (5)	0.3700 (5)	0.5884 (5)	0.094 (3)	
C4	1.1859 (3)	0.3338 (3)	1.1067 (5)	0.0657 (17)	
H4	1.2356	0.3236	1.1221	0.079*	
C42	0.8206 (3)	0.4439 (3)	1.2886 (4)	0.0717 (18)	
H42	0.7890	0.4843	1.2757	0.086*	
C17	0.8225 (4)	0.0940 (4)	0.6520 (5)	0.092 (2)	
H17	0.7999	0.1406	0.6613	0.111*	
C37	0.4615 (3)	0.3164 (4)	1.4366 (4)	0.0707 (17)	
H37	0.4664	0.2816	1.4846	0.085*	
C44	0.9278 (3)	0.3955 (5)	1.3627 (4)	0.076 (2)	
C27	0.8582 (3)	0.4651 (4)	0.8466 (5)	0.0771 (19)	
H27	0.8727	0.5123	0.8253	0.093*	
C23	1.1331 (4)	0.2674 (4)	0.6902 (4)	0.086 (2)	
H23	1.1485	0.2217	0.7152	0.103*	
C15	0.8461 (5)	−0.0133 (5)	0.5573 (6)	0.106 (3)	
H15	0.8401	−0.0395	0.5016	0.127*	
C2	1.0653 (4)	0.3787 (4)	1.1482 (5)	0.084 (2)	
H2	1.0330	0.3982	1.1930	0.101*	
C22	1.1767 (4)	0.3036 (5)	0.6216 (5)	0.106 (3)	
H22	1.2213	0.2826	0.5999	0.128*	
C53	1.0402 (3)	0.3691 (3)	1.0587 (5)	0.0789 (18)	
H53	0.9912	0.3817	1.0423	0.095*	
C14	0.8885 (5)	−0.0432 (5)	0.6237 (6)	0.121 (3)	
H14	0.9112	−0.0897	0.6144	0.145*	
C20	1.0880 (5)	0.4040 (4)	0.6183 (5)	0.096 (2)	
H20	1.0729	0.4502	0.5945	0.115*	
C51	0.8501 (4)	0.3657 (4)	0.6251 (5)	0.106 (2)	
H51A	0.8833	0.3242	0.6369	0.159*	
H51B	0.8593	0.3851	0.5636	0.159*	
H51C	0.7989	0.3490	0.6295	0.159*	
C50	1.2292 (4)	0.3516 (4)	1.2947 (4)	0.112 (3)	
H50A	1.2366	0.2984	1.2880	0.168*	
H50B	1.2357	0.3655	1.3589	0.168*	
H50C	1.2652	0.3779	1.2570	0.168*	

### Atomic displacement parameters (Å^2^)

**Table:**

	*U*^11^	*U*^22^	*U*^33^	*U*^12^	*U*^13^	*U*^23^
C31	0.042 (3)	0.046 (3)	0.035 (3)	−0.001 (2)	0.004 (3)	−0.002 (3)
O2	0.053 (2)	0.093 (3)	0.042 (3)	−0.005 (2)	−0.0041 (19)	−0.003 (2)
N7	0.037 (3)	0.052 (3)	0.034 (3)	−0.003 (2)	0.000 (2)	−0.002 (2)
C28	0.046 (4)	0.044 (3)	0.043 (4)	−0.001 (3)	0.002 (3)	0.003 (3)
N9	0.044 (3)	0.067 (3)	0.032 (3)	0.002 (2)	0.000 (2)	−0.003 (2)
N8	0.039 (3)	0.043 (2)	0.044 (3)	0.001 (2)	−0.003 (2)	−0.004 (2)
N4	0.046 (3)	0.062 (3)	0.041 (3)	−0.009 (2)	0.002 (2)	0.008 (2)
O3	0.036 (2)	0.081 (3)	0.049 (3)	−0.0028 (18)	−0.0016 (18)	0.004 (2)
O4	0.069 (3)	0.069 (3)	0.054 (3)	−0.028 (2)	−0.005 (2)	0.006 (2)
O1	0.094 (3)	0.103 (3)	0.039 (2)	−0.033 (2)	0.008 (2)	0.001 (2)
C34	0.041 (3)	0.088 (5)	0.046 (4)	−0.007 (3)	−0.004 (3)	−0.007 (4)
N10	0.056 (3)	0.106 (4)	0.062 (4)	−0.012 (3)	0.001 (3)	−0.005 (3)
C26	0.048 (4)	0.071 (5)	0.064 (5)	0.010 (3)	0.018 (3)	0.015 (4)
C5	0.044 (3)	0.057 (4)	0.053 (4)	−0.004 (3)	−0.003 (3)	0.002 (3)
N5	0.067 (3)	0.054 (3)	0.049 (3)	−0.007 (2)	0.005 (3)	−0.003 (3)
C6	0.054 (4)	0.060 (4)	0.049 (4)	−0.002 (3)	−0.005 (3)	0.004 (3)
C11	0.067 (4)	0.056 (4)	0.058 (4)	−0.022 (3)	0.015 (4)	−0.010 (3)
C24	0.038 (3)	0.055 (4)	0.054 (4)	0.004 (3)	0.006 (3)	0.012 (3)
C7	0.051 (3)	0.054 (4)	0.045 (4)	−0.009 (3)	0.006 (3)	0.009 (3)
C1	0.045 (4)	0.058 (4)	0.051 (4)	−0.010 (3)	0.010 (3)	0.003 (3)
C25	0.054 (4)	0.068 (4)	0.059 (4)	0.012 (3)	0.005 (3)	−0.006 (4)
N3	0.044 (3)	0.050 (3)	0.048 (3)	−0.009 (2)	0.001 (2)	0.001 (2)
C33	0.042 (3)	0.063 (4)	0.031 (3)	−0.006 (3)	0.001 (3)	−0.004 (3)
C30	0.040 (3)	0.043 (3)	0.040 (4)	0.001 (3)	−0.002 (3)	0.002 (3)
C29	0.044 (4)	0.055 (4)	0.039 (4)	0.000 (3)	−0.007 (3)	−0.004 (3)
C55	0.068 (4)	0.067 (4)	0.059 (4)	−0.009 (3)	0.013 (3)	0.002 (3)
O5	0.108 (4)	0.105 (4)	0.064 (3)	−0.018 (3)	−0.002 (3)	−0.024 (3)
O6	0.093 (3)	0.108 (4)	0.064 (3)	0.006 (3)	0.024 (3)	0.027 (3)
C40	0.085 (5)	0.096 (5)	0.046 (4)	0.015 (4)	0.005 (4)	0.004 (4)
F2	0.081 (3)	0.168 (4)	0.104 (3)	−0.031 (3)	−0.054 (2)	0.022 (3)
C18	0.049 (4)	0.072 (4)	0.046 (4)	−0.012 (3)	−0.010 (3)	0.010 (3)
N6	0.112 (5)	0.122 (5)	0.057 (4)	−0.044 (4)	0.024 (4)	−0.014 (4)
C32	0.046 (4)	0.062 (4)	0.058 (4)	−0.005 (3)	−0.007 (3)	−0.004 (3)
C12	0.051 (4)	0.063 (4)	0.047 (4)	−0.009 (3)	0.009 (3)	−0.009 (3)
C41	0.040 (3)	0.050 (3)	0.045 (3)	0.005 (3)	−0.007 (3)	0.003 (3)
C35	0.039 (3)	0.064 (4)	0.038 (4)	0.002 (3)	0.002 (3)	−0.004 (3)
C3	0.070 (5)	0.059 (4)	0.069 (5)	−0.018 (3)	0.009 (4)	−0.014 (4)
C10	0.060 (4)	0.048 (4)	0.047 (4)	−0.011 (3)	0.011 (3)	−0.004 (3)
C13	0.114 (6)	0.094 (5)	0.065 (5)	0.015 (5)	−0.019 (4)	−0.025 (4)
C16	0.107 (6)	0.127 (7)	0.064 (6)	−0.033 (6)	−0.026 (5)	0.040 (6)
C8	0.047 (3)	0.064 (4)	0.034 (3)	0.004 (3)	0.008 (3)	−0.002 (3)
C52	0.064 (4)	0.063 (4)	0.054 (4)	−0.007 (3)	0.003 (3)	−0.006 (3)
C45	0.066 (5)	0.095 (6)	0.104 (6)	0.008 (4)	−0.036 (4)	0.030 (5)
C19	0.083 (5)	0.072 (4)	0.065 (4)	−0.023 (4)	−0.008 (4)	0.017 (4)
F1	0.172 (4)	0.208 (5)	0.061 (3)	−0.120 (4)	0.008 (3)	0.020 (3)
C36	0.072 (4)	0.068 (4)	0.047 (4)	0.006 (3)	0.005 (3)	−0.005 (3)
C9	0.072 (4)	0.059 (4)	0.046 (4)	−0.010 (3)	0.021 (3)	0.008 (3)
C38	0.066 (5)	0.120 (6)	0.053 (5)	−0.011 (4)	0.015 (4)	−0.019 (5)
C39	0.086 (5)	0.123 (6)	0.060 (5)	0.025 (4)	0.013 (4)	−0.012 (5)
C46	0.068 (4)	0.053 (4)	0.074 (4)	0.003 (3)	−0.024 (4)	0.003 (3)
C43	0.087 (5)	0.077 (5)	0.080 (5)	−0.016 (4)	−0.021 (4)	−0.018 (4)
C54	0.051 (4)	0.052 (4)	0.064 (4)	0.006 (3)	−0.003 (3)	0.005 (3)
C21	0.087 (6)	0.151 (8)	0.045 (5)	−0.077 (6)	0.003 (4)	0.019 (5)
C4	0.058 (4)	0.062 (4)	0.077 (5)	−0.001 (3)	−0.005 (4)	−0.007 (4)
C42	0.068 (4)	0.062 (4)	0.086 (5)	0.003 (3)	−0.025 (4)	−0.020 (4)
C17	0.115 (6)	0.079 (5)	0.082 (5)	−0.008 (4)	−0.030 (5)	0.020 (4)
C37	0.080 (5)	0.080 (5)	0.052 (4)	−0.010 (4)	0.009 (4)	0.007 (4)
C44	0.053 (4)	0.109 (6)	0.065 (5)	−0.023 (4)	−0.023 (4)	0.013 (5)
C27	0.088 (5)	0.061 (4)	0.082 (5)	−0.010 (4)	0.019 (4)	0.014 (4)
C23	0.072 (5)	0.116 (6)	0.070 (5)	0.002 (4)	0.020 (4)	0.025 (4)
C15	0.113 (7)	0.145 (9)	0.058 (5)	−0.034 (6)	0.014 (5)	0.001 (6)
C2	0.088 (6)	0.102 (5)	0.062 (5)	−0.006 (4)	0.022 (4)	−0.015 (4)
C22	0.068 (5)	0.181 (9)	0.070 (6)	−0.018 (5)	0.012 (4)	0.020 (6)
C53	0.048 (4)	0.095 (5)	0.094 (5)	0.003 (3)	0.010 (4)	−0.007 (4)
C14	0.149 (8)	0.118 (7)	0.095 (7)	0.020 (6)	−0.014 (6)	−0.047 (6)
C20	0.111 (6)	0.085 (5)	0.091 (6)	−0.044 (5)	−0.012 (5)	0.030 (5)
C51	0.124 (6)	0.133 (7)	0.062 (5)	0.027 (5)	0.006 (5)	−0.005 (5)
C50	0.145 (7)	0.126 (6)	0.066 (5)	−0.010 (6)	−0.023 (5)	−0.004 (5)

### Geometric parameters (Å, °)

**Table:**

C31—N8	1.374 (5)	C41—C46	1.356 (6)
C31—C30	1.386 (6)	C41—C42	1.369 (7)
C31—C33	1.420 (6)	C35—C36	1.365 (7)
O2—C29	1.225 (5)	C3—C4	1.359 (7)
N7—C28	1.384 (6)	C3—C2	1.379 (8)
N7—C29	1.420 (6)	C10—C9	1.381 (7)
N7—C41	1.444 (6)	C10—C8	1.423 (6)
C28—N8	1.279 (5)	C13—C14	1.389 (8)
C28—O3	1.351 (5)	C13—H13	0.9300
N9—C32	1.341 (6)	C16—C15	1.355 (9)
N9—C30	1.396 (5)	C16—C17	1.422 (9)
N9—C35	1.443 (6)	C16—H16	0.9300
N4—C5	1.379 (6)	C52—C4	1.378 (7)
N4—C6	1.422 (6)	C52—H52	0.9300
N4—C18	1.433 (6)	C45—C44	1.357 (8)
O3—C24	1.420 (6)	C45—C46	1.367 (7)
O4—C5	1.344 (5)	C45—H45	0.9300
O4—C1	1.422 (6)	C19—C20	1.358 (8)
O4—C55	4.581 (6)	C19—H19	0.9300
O1—C6	1.204 (6)	F1—C21	1.374 (7)
C34—N10	1.150 (6)	C36—C37	1.382 (7)
C34—C33	1.398 (7)	C36—H36	0.9300
C26—C25	1.369 (7)	C9—H9	0.9300
C26—O6	1.375 (6)	C38—C39	1.357 (8)
C26—C27	1.379 (7)	C38—C37	1.367 (7)
C5—N3	1.275 (6)	C38—H38	0.9300
N5—C9	1.348 (6)	C39—H39	0.9300
N5—C7	1.393 (6)	C46—H46	0.9300
N5—C12	1.455 (6)	C43—C44	1.322 (8)
C6—C7	1.433 (7)	C43—C42	1.398 (7)
C11—N6	1.135 (6)	C43—H43	0.9300
C11—C10	1.433 (7)	C54—H54	0.9300
C24—C54	1.354 (7)	C21—C22	1.352 (10)
C24—C55	1.357 (7)	C21—C20	1.347 (10)
C7—C8	1.377 (6)	C4—H4	0.9300
C1—C52	1.347 (7)	C42—H42	0.9300
C1—C53	1.390 (7)	C17—H17	0.9300
C25—C54	1.378 (7)	C37—H37	0.9300
C25—H25	0.9300	C27—H27	0.9300
N3—C8	1.358 (6)	C23—C22	1.409 (8)
C33—C32	1.389 (6)	C23—H23	0.9300
C30—C29	1.424 (6)	C15—C14	1.327 (9)
C55—C27	1.381 (7)	C15—H15	0.9300
C55—H55	0.9300	C2—C53	1.370 (8)
O5—C3	1.378 (7)	C2—H2	0.9300
O5—C50	1.412 (7)	C22—H22	0.9300
O6—C51	1.419 (6)	C53—H53	0.9300
C40—C35	1.362 (7)	C14—H14	0.9300
C40—C39	1.363 (7)	C20—H20	0.9300
C40—H40	0.9300	C51—H51A	0.9600
F2—C44	1.356 (6)	C51—H51B	0.9600
C18—C19	1.354 (7)	C51—H51C	0.9600
C18—C23	1.363 (7)	C50—H50A	0.9600
C32—H32	0.9300	C50—H50B	0.9600
C12—C13	1.353 (7)	C50—H50C	0.9600
C12—C17	1.358 (7)		
			
N8—C31—C30	124.2 (5)	C15—C16—C17	117.9 (8)
N8—C31—C33	127.4 (4)	C15—C16—H16	121.1
C30—C31—C33	108.4 (4)	C17—C16—H16	121.0
C28—N7—C29	122.3 (4)	N3—C8—C7	124.2 (5)
C28—N7—C41	121.2 (4)	N3—C8—C10	129.9 (5)
C29—N7—C41	116.2 (4)	C7—C8—C10	105.9 (5)
N8—C28—O3	122.2 (5)	C1—C52—C4	120.5 (5)
N8—C28—N7	126.4 (5)	C1—C52—H52	119.7
O3—C28—N7	111.4 (5)	C4—C52—H52	119.7
C32—N9—C30	108.4 (4)	C44—C45—C46	119.7 (6)
C32—N9—C35	124.2 (4)	C44—C45—H45	120.2
C30—N9—C35	127.2 (4)	C46—C45—H45	120.1
C28—N8—C31	114.0 (4)	C18—C19—C20	122.4 (7)
C5—N4—C6	121.7 (5)	C18—C19—H19	118.8
C5—N4—C18	122.5 (5)	C20—C19—H19	118.8
C6—N4—C18	115.7 (4)	C35—C36—C37	119.4 (5)
C28—O3—C24	116.6 (4)	C35—C36—H36	120.3
C5—O4—C1	117.4 (4)	C37—C36—H36	120.3
C5—O4—C55	82.0 (3)	N5—C9—C10	109.7 (5)
C1—O4—C55	93.1 (3)	N5—C9—H9	125.1
N10—C34—C33	177.2 (7)	C10—C9—H9	125.1
C25—C26—O6	125.0 (6)	C39—C38—C37	118.7 (6)
C25—C26—C27	118.6 (6)	C39—C38—H38	120.6
O6—C26—C27	116.4 (6)	C37—C38—H38	120.6
N3—C5—O4	122.7 (5)	C38—C39—C40	121.3 (6)
N3—C5—N4	126.9 (5)	C38—C39—H39	119.4
O4—C5—N4	110.4 (5)	C40—C39—H39	119.3
C9—N5—C7	107.6 (5)	C41—C46—C45	119.8 (6)
C9—N5—C12	125.6 (5)	C41—C46—H46	120.1
C7—N5—C12	126.7 (5)	C45—C46—H46	120.1
O1—C6—N4	120.8 (5)	C44—C43—C42	119.2 (6)
O1—C6—C7	128.5 (5)	C44—C43—H43	120.4
N4—C6—C7	110.7 (5)	C42—C43—H43	120.4
N6—C11—C10	177.0 (7)	C24—C54—C25	119.7 (6)
C54—C24—C55	120.9 (6)	C24—C54—H54	120.2
C54—C24—O3	120.7 (5)	C25—C54—H54	120.2
C55—C24—O3	118.3 (5)	C22—C21—C20	124.1 (7)
C8—C7—N5	109.4 (5)	C22—C21—F1	116.8 (9)
C8—C7—C6	122.0 (5)	C20—C21—F1	119.2 (9)
N5—C7—C6	128.5 (5)	C3—C4—C52	119.9 (6)
C52—C1—C53	120.7 (6)	C3—C4—H4	120.0
C52—C1—O4	118.1 (5)	C52—C4—H4	120.0
C53—C1—O4	121.2 (6)	C41—C42—C43	119.4 (6)
C26—C25—C54	120.7 (6)	C41—C42—H42	120.3
C26—C25—H25	119.6	C43—C42—H42	120.3
C54—C25—H25	119.6	C12—C17—C16	118.8 (7)
C5—N3—C8	114.4 (5)	C12—C17—H17	120.6
C32—C33—C34	127.8 (5)	C16—C17—H17	120.6
C32—C33—C31	105.1 (4)	C38—C37—C36	120.7 (6)
C34—C33—C31	127.0 (5)	C38—C37—H37	119.7
C31—C30—N9	107.2 (4)	C36—C37—H37	119.7
C31—C30—C29	121.9 (5)	C43—C44—F2	119.0 (7)
N9—C30—C29	130.8 (5)	C43—C44—C45	121.8 (6)
O2—C29—C30	128.5 (5)	F2—C44—C45	119.1 (7)
O2—C29—N7	120.6 (5)	C26—C27—C55	120.4 (6)
C30—C29—N7	110.8 (4)	C26—C27—H27	119.8
C24—C55—C27	119.6 (6)	C55—C27—H27	119.8
C24—C55—O4	80.3 (3)	C18—C23—C22	119.2 (7)
C27—C55—O4	77.6 (4)	C18—C23—H23	120.4
C24—C55—H55	120.2	C22—C23—H23	120.4
C27—C55—H55	120.2	C14—C15—C16	122.5 (9)
O4—C55—H55	112.3	C14—C15—H15	118.8
C3—O5—C50	117.8 (5)	C16—C15—H15	118.7
C26—O6—C51	117.9 (5)	C53—C2—C3	121.1 (6)
C35—C40—C39	120.0 (6)	C53—C2—H2	119.4
C35—C40—H40	120.0	C3—C2—H2	119.4
C39—C40—H40	120.0	C21—C22—C23	117.4 (7)
C19—C18—C23	119.8 (6)	C21—C22—H22	121.3
C19—C18—N4	121.7 (6)	C23—C22—H22	121.3
C23—C18—N4	118.4 (5)	C2—C53—C1	118.2 (6)
N9—C32—C33	110.8 (4)	C2—C53—H53	120.9
N9—C32—H32	124.6	C1—C53—H53	120.9
C33—C32—H32	124.6	C15—C14—C13	120.1 (8)
C13—C12—C17	121.6 (6)	C15—C14—H14	119.9
C13—C12—N5	116.7 (6)	C13—C14—H14	120.0
C17—C12—N5	121.7 (6)	C21—C20—C19	117.1 (7)
C46—C41—C42	120.0 (5)	C21—C20—H20	121.4
C46—C41—N7	120.6 (5)	C19—C20—H20	121.4
C42—C41—N7	119.4 (5)	O6—C51—H51A	109.5
C40—C35—C36	119.9 (5)	O6—C51—H51B	109.5
C40—C35—N9	118.9 (5)	H51A—C51—H51B	109.5
C36—C35—N9	121.1 (5)	O6—C51—H51C	109.5
C4—C3—C2	119.5 (6)	H51A—C51—H51C	109.5
C4—C3—O5	125.0 (7)	H51B—C51—H51C	109.5
C2—C3—O5	115.5 (6)	O5—C50—H50A	109.5
C9—C10—C8	107.3 (5)	O5—C50—H50B	109.5
C9—C10—C11	127.3 (5)	H50A—C50—H50B	109.5
C8—C10—C11	125.2 (5)	O5—C50—H50C	109.5
C12—C13—C14	119.0 (7)	H50A—C50—H50C	109.5
C12—C13—H13	120.5	H50B—C50—H50C	109.5
C14—C13—H13	120.5		
			
C29—N7—C28—N8	−8.1 (8)	C7—N5—C12—C13	101.5 (7)
C41—N7—C28—N8	178.7 (5)	C9—N5—C12—C17	108.7 (7)
C29—N7—C28—O3	172.7 (4)	C7—N5—C12—C17	−76.0 (8)
C41—N7—C28—O3	−0.6 (6)	C28—N7—C41—C46	−79.3 (6)
O3—C28—N8—C31	−176.0 (4)	C29—N7—C41—C46	107.1 (5)
N7—C28—N8—C31	4.8 (7)	C28—N7—C41—C42	99.9 (6)
C30—C31—N8—C28	−0.9 (7)	C29—N7—C41—C42	−73.8 (6)
C33—C31—N8—C28	176.9 (5)	C39—C40—C35—C36	0.2 (9)
N8—C28—O3—C24	8.7 (7)	C39—C40—C35—N9	178.4 (5)
N7—C28—O3—C24	−172.0 (4)	C32—N9—C35—C40	−58.7 (7)
C1—O4—C5—N3	14.3 (7)	C30—N9—C35—C40	114.5 (6)
C55—O4—C5—N3	−75.1 (5)	C32—N9—C35—C36	119.4 (6)
C1—O4—C5—N4	−164.6 (4)	C30—N9—C35—C36	−67.4 (7)
C55—O4—C5—N4	106.0 (4)	C50—O5—C3—C4	−1.5 (9)
C6—N4—C5—N3	1.1 (8)	C50—O5—C3—C2	179.5 (6)
C18—N4—C5—N3	−175.5 (5)	C17—C12—C13—C14	2.2 (10)
C6—N4—C5—O4	179.9 (4)	N5—C12—C13—C14	−175.4 (6)
C18—N4—C5—O4	3.3 (7)	C5—N3—C8—C7	1.7 (7)
C5—N4—C6—O1	177.9 (5)	C5—N3—C8—C10	−176.8 (5)
C18—N4—C6—O1	−5.2 (8)	N5—C7—C8—N3	−179.2 (4)
C5—N4—C6—C7	−0.5 (7)	C6—C7—C8—N3	−1.3 (8)
C18—N4—C6—C7	176.3 (4)	N5—C7—C8—C10	−0.5 (6)
C28—O3—C24—C54	−74.4 (6)	C6—C7—C8—C10	177.5 (5)
C28—O3—C24—C55	109.8 (5)	C9—C10—C8—N3	179.1 (5)
C9—N5—C7—C8	0.3 (6)	C11—C10—C8—N3	3.0 (9)
C12—N5—C7—C8	−175.6 (5)	C9—C10—C8—C7	0.5 (6)
C9—N5—C7—C6	−177.5 (5)	C11—C10—C8—C7	−175.7 (5)
C12—N5—C7—C6	6.6 (9)	C53—C1—C52—C4	−0.6 (8)
O1—C6—C7—C8	−177.6 (6)	O4—C1—C52—C4	177.1 (5)
N4—C6—C7—C8	0.6 (7)	C23—C18—C19—C20	2.2 (9)
O1—C6—C7—N5	−0.2 (10)	N4—C18—C19—C20	−174.4 (5)
N4—C6—C7—N5	178.1 (5)	C40—C35—C36—C37	0.0 (8)
C5—O4—C1—C52	107.7 (5)	N9—C35—C36—C37	−178.1 (5)
C55—O4—C1—C52	−169.8 (4)	C12—N5—C9—C10	176.0 (5)
C5—O4—C1—C53	−74.7 (6)	C8—C10—C9—N5	−0.3 (6)
C55—O4—C1—C53	7.9 (5)	C11—C10—C9—N5	175.7 (5)
O6—C26—C25—C54	−179.6 (5)	C37—C38—C39—C40	−0.6 (10)
C27—C26—C25—C54	0.8 (8)	C42—C41—C46—C45	3.3 (9)
O4—C5—N3—C8	179.7 (4)	N7—C41—C46—C45	−177.5 (5)
N4—C5—N3—C8	−1.6 (8)	C44—C45—C46—C41	−1.1 (10)
N8—C31—C33—C32	−177.0 (4)	C55—C24—C54—C25	−2.5 (8)
C30—C31—C33—C32	1.1 (6)	O3—C24—C54—C25	−178.1 (4)
N8—C31—C33—C34	3.6 (9)	C26—C25—C54—C24	1.5 (8)
C30—C31—C33—C34	−178.4 (5)	C2—C3—C4—C52	−3.5 (9)
N8—C31—C30—N9	176.9 (4)	O5—C3—C4—C52	177.5 (5)
C33—C31—C30—N9	−1.2 (5)	C1—C52—C4—C3	2.7 (9)
N8—C31—C30—C29	0.1 (8)	C46—C41—C42—C43	−2.9 (8)
C33—C31—C30—C29	−178.0 (4)	N7—C41—C42—C43	177.9 (5)
C32—N9—C30—C31	0.9 (5)	C44—C43—C42—C41	0.2 (9)
C35—N9—C30—C31	−173.2 (5)	C13—C12—C17—C16	−1.2 (10)
C32—N9—C30—C29	177.3 (5)	N5—C12—C17—C16	176.3 (5)
C35—N9—C30—C29	3.2 (8)	C15—C16—C17—C12	−0.8 (11)
C31—C30—C29—O2	178.4 (5)	C39—C38—C37—C36	0.9 (9)
N9—C30—C29—O2	2.5 (9)	C35—C36—C37—C38	−0.6 (9)
C31—C30—C29—N7	−2.6 (7)	C42—C43—C44—F2	−178.9 (5)
N9—C30—C29—N7	−178.6 (4)	C42—C43—C44—C45	2.1 (10)
C28—N7—C29—O2	−174.8 (5)	C46—C45—C44—C43	−1.7 (11)
C41—N7—C29—O2	−1.2 (7)	C46—C45—C44—F2	179.3 (5)
C28—N7—C29—C30	6.2 (6)	C25—C26—C27—C55	−2.2 (9)
C41—N7—C29—C30	179.7 (4)	O6—C26—C27—C55	178.2 (5)
C54—C24—C55—C27	1.1 (8)	C24—C55—C27—C26	1.3 (9)
O3—C24—C55—C27	176.8 (5)	O4—C55—C27—C26	72.5 (5)
C54—C24—C55—O4	−68.6 (5)	C19—C18—C23—C22	−2.0 (9)
O3—C24—C55—O4	107.1 (4)	N4—C18—C23—C22	174.8 (5)
C5—O4—C55—C24	34.7 (4)	C17—C16—C15—C14	1.8 (13)
C1—O4—C55—C24	−82.6 (4)	C4—C3—C2—C53	2.3 (10)
C5—O4—C55—C27	−88.7 (5)	O5—C3—C2—C53	−178.6 (6)
C1—O4—C55—C27	154.0 (4)	C20—C21—C22—C23	1.3 (11)
C25—C26—O6—C51	5.2 (8)	F1—C21—C22—C23	−177.7 (5)
C27—C26—O6—C51	−175.2 (5)	C18—C23—C22—C21	0.3 (10)
C5—N4—C18—C19	−84.3 (7)	C3—C2—C53—C1	−0.2 (10)
C6—N4—C18—C19	98.9 (6)	C52—C1—C53—C2	−0.6 (9)
C5—N4—C18—C23	99.0 (7)	O4—C1—C53—C2	−178.2 (5)
C6—N4—C18—C23	−77.8 (6)	C16—C15—C14—C13	−0.9 (14)
C30—N9—C32—C33	−0.2 (6)	C12—C13—C14—C15	−1.2 (12)
C35—N9—C32—C33	174.1 (4)	C22—C21—C20—C19	−1.2 (11)
C34—C33—C32—N9	178.9 (5)	F1—C21—C20—C19	177.8 (5)
C31—C33—C32—N9	−0.6 (6)	C18—C19—C20—C21	−0.6 (9)
C9—N5—C12—C13	−73.7 (7)		

### Hydrogen-bond geometry (Å, °)

**Table:**

*D*—H···*A*	*D*—H	H···*A*	*D*···*A*	*D*—H···*A*
C19—H19···O6	0.93	2.57	3.421 (8)	152.
C38—H38···O2^i^	0.93	2.61	3.405 (8)	144.
C50—H50B···F1^ii^	0.96	2.51	3.407 (7)	155.
C15—H15···O1^iii^	0.93	2.41	3.201 (9)	143.

Symmetry codes: (i) *y*, −*x*+1, −*z*+3; (ii) *x*, *y*, *z*+1; (iii) −*y*+1, *x*−1, −*z*+1.

## References

[bb1] Bruker (2001). *SMART* and *SAINT-Plus* Bruker AXS Inc., Madison, Wisconsin, USA.

[bb2] Ding, M. W., Xu, S. Z. & Zhao, J. F. (2004). *J. Org. Chem.* **69**, 8366–8371. 10.1021/jo048691v 15549808

[bb3] Niwas, S., Chand, P., Pathak, V. P. & Montgomery, J. A. (1994). *J. Med. Chem.* **37**, 2477–2480. 10.1021/jm00041a027 8057293

[bb4] Sheldrick, G. M. (2003). *SADABS* Bruker AXS Inc., Madison, Wisconsin, USA.

[bb5] Sheldrick, G. M. (2008). *Acta Cryst.* A**64**, 112–122. 10.1107/S0108767307043930 18156677

[bb6] Shih, H., Cottam, H. B. & Carson, D. A. (2002). *Chem. Pharm. Bull.* **50**, 364–367. 10.1248/cpb.50.364 11911199

[bb7] Spek, A. L. (2003). *J. Appl. Cryst.* **36**, 7–13.

